# Supplementary material for: Metabolomics and Cytokine Signatures in COVID-19: Uncovering Immunometabolism in Pathogenesis
Source: Metabolites. 2025 Sep 11;15(9):608. doi: 10.3390/metabo15090608 (PMC12472153; doi:10.3390/metabo15090608)
Supplement: Supplementary file 1 [file metabolites-15-00608-s001.zip › metabolites-3827810-supplementary.pdf]

Metabolomics and Cytokine Signatures in COVID-19: Uncovering the immunometabolism in the pathogenesis

Mohammad Mehdi Banoei[1,2], Abdulrazagh Hashemi Shahraki[3], Kayo Santos[4], Gergory Holt[4], Mehdi Mirsaeidi[3†]

1 Department of Critical Care Medicine, University of Calgary, Calgary, AB, T2N 4Z6, Canada.

2 Department of Biomedical Engineering, Schulich School of Engineering, University of Calgary, Calgary, AB, T2N 1N4, Canada. mmbanoei@ucalgary.ca (M.M.B.)

3 Division of Pulmonary, Critical Care, and Sleep, College of Medicine - Jacksonville, University of Florida, Jacksonville, FL, 32209, USA, hashemishahrak.a@ufl.edu (A.H.S.)

4 Division of Pulmonary and Critical Care, University of Miami, Miami, FL, 33146, USA, kayohenrique.md@gmail.com (K.S.); gholt@miami.edu (G.H.)

[†] Corresponding Author:

Mehdi Mirsaeidi, M.D., M.P.H.,

Division of Pulmonary, Critical Care, and Sleep Medicine,

College of Medicine – Jacksonville, University of Florida,

653-1 8th Street West, Jacksonville, FL 32209. USA

E-mail: m.mirsaeidi@ufl.edu

Correlation between metabolomics and cytokine profiling, an overview

We observed a much higher correlation between IL-1β, IL-2, IFN-β, IFN-γ, IL-17, and GM-CSF inflammatory markers and metabolites (Fig. S2). Different metabolite-cytokine correlation patterns were observed concerning COVID-19 mortality (Figs. S2). The results showed that metabolites such as 3-hydroxykynurenine, urocanate, paraxanthine, serotonin, LysoPCs (14:0, 16:0, 17:0, and 18:2), acylcarnitines (C14:0, C16:0, C18:0, C18:1, and C18:2), 5-hydroxyisourate, N4-acetylspermine, lysophosphatidic acid, kynurenic acid, urate, and ethylmalonic acid had higher positive correlations with IL-1β, IL-2, IFN-β, IFN-γ, IL-17, and GM-CSF in COVID-19 non-survivors than in COVID-19 survivors (Fig. S2), indicating the role of related metabolism and immunometabolism pathway impact on mortality outcome. In addition, picolinic acid, quinate, methylhistidine, nicotinate, and N-acetyl-glycine showed a high negative correlation with cytokine levels, which was linked to the worst outcome. Pyruvate and inosine showed significantly higher correlations with cytokines among non-survivors than survivors. Among COVID-19 survivors, serine, hypotaurine, S-methylcysteine, N-formylglycine, D-sorbitol, aconitic, and hypoxanthine had considerably higher correlations with cytokines than non-survivors (Fig. S2).

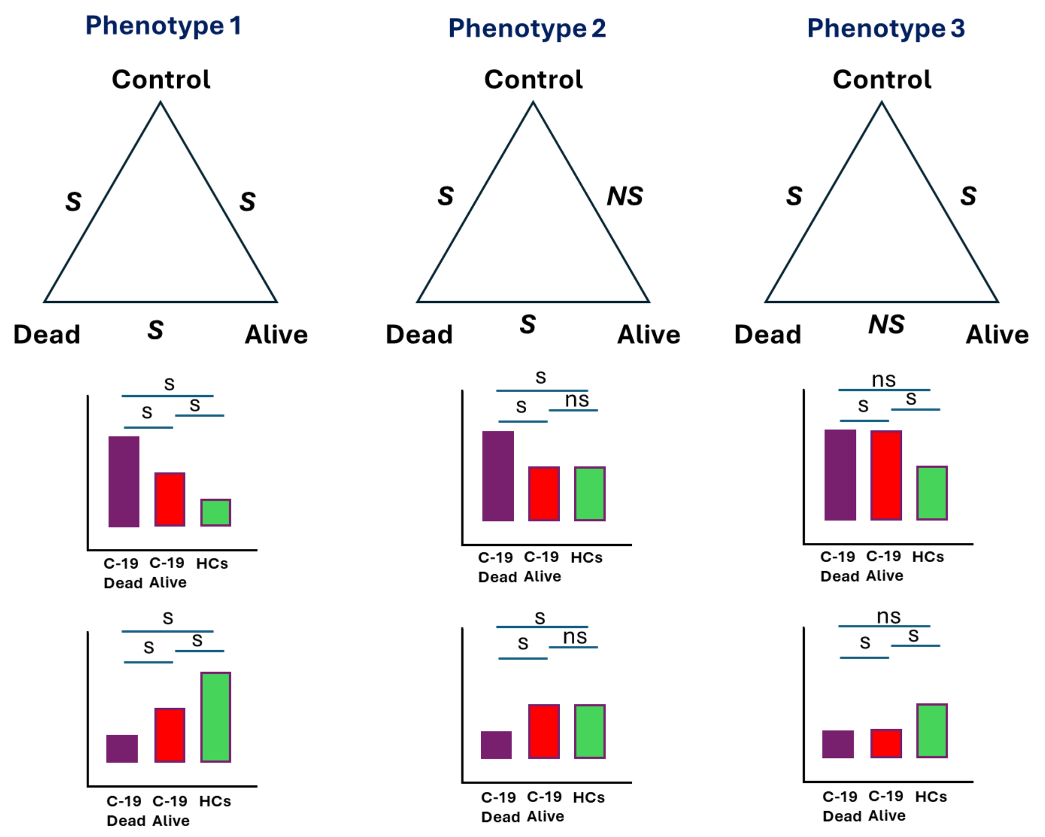

Figure S1. The figure shows that different metabolic phenotypes were described based on the significant and non-significant changes in metabolites among three COVID-19 dead, COVID-19 alive and healthy control cohorts.

Name	Mean (SD) of Survivors	Mean (SD) of Non-survivors	p-value	Fold Change	Non-survivors vs. Survivors
IL-6*	17.575 (15.41)	64.608 (76.60)	0.0063	3.68	Up
TNF alpha*	3.055 (0.59)	5.627 (6.56)	0.0021	1.84	Up
IL-2R	4840.41 (7110.23)	8157.71 (11615.20)	0.4135	1.69	Up
IL 10	1.806 (1.25)	2.792 (2.62)	0.7352	1.55	Up
IL-8(CXCL8)	10.325 (10.93)	15.739 (16.83)	0.4249	1.52	Up
IL-7	2.306 (1.43)	2.885 (1.83)	0.2615	1.25	Up
IFN gamma*	2.623 (1.06)	2.845 (0.75)	0.0489	1.08	Up
IL-12/IL-23p40	1.188 (1.71)	1.286 (2.20)	0.1700	1.08	Up
IL-1 Beta	1.136 (0.10)	1.206 (0.15)	0.1279	1.06	Up
IL-2*	2.204 (1.69)	2.040 (0.27)	0.0276	-1.08	Down
IFN alpha	1.147 (2.18)	1.054 (2.54)	0.4061	-1.09	Down
GM-CSF	5.579 (2.49)	5.051 (0.51)	0.7189	-1.1	Down
IL-17A(CTLA-8)	1.480 (2.10)	1.089 (0.16)	0.1500	-1.36	Down
IFN beta	3.473 (8.44)	1.558 (0.66)	0.5877	-2.23	Down

Table S1. The table shows the differences in 14 cytokines between COVID-19 non-survivors and survivors.

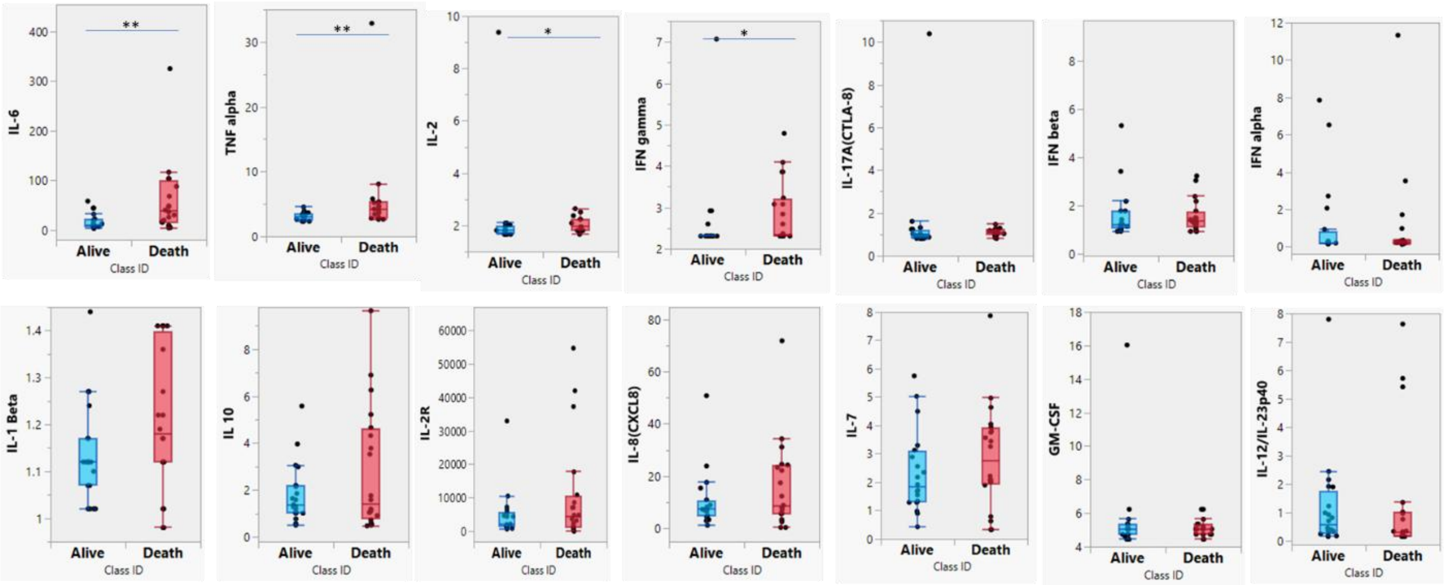

Figure S2, Boxplots showing the difference in 14 cytokines among survivors (alive) and non-survivors (dead).

	COVID-19 Mortality Outcome		
	Phenotype 1	Phenotype 2	Phenotype 3
1	L-ALANINE	N-FORMYLGLYCINE	AZELAIC ACID
2	5-OXO-D-PROLINE	METHYLMALONATE	N-ACETYL-D-GALACTOSAMINE
3	N-ACETYL-L-ALANINE	L-ISOLEUCINE	CARNOSINE
4	ETHYLMALONIC ACID	XANTHOSINE	ACETOACETATE
5	3-UREIDOPROPIONATE	N-AMIDINO-L-ASPARTATE	3-HYDROXYBUTANOIC ACID
6	4-ACETAMIDOBUTANOATE	PHENYLACETALGLUTAMINE	HYPOTAURINE
7	N-ACETYL-DL-SERINE	C5-DC (O-glutanoyl-L-carntine)	N-ACETYLGLYCINE
8	XANTHINE	C5-OH	ITACONATE
9	D-GLUCURONIC ACID	C10:1	GLUTARATE
10	URIDINE	2-OXOBUTANOATE	L-ASPARTATE
11	5-HYDROXY-L-TRYPTOPHAN	ALLOSE	3-HYDROXYBENZOATE
12	KYNURENINE	ALPHA-D-GLUCOSE	L-GLUTAMIC ACID
13	C5-DC (O-glutarylL-carntine)	ADENINE	6-CARBOXYHEXANOATE
14	C6 DC	N-METHYL-L-GLUTAMATE	DL-5-HYDROXYLYSINE
15	L-CYSTATHIONINE	CHORISMATE	L-ARGININE
16	ACETYLSPERMINE	MALIC ACID	SUBERIC ACID
17	LysoPC 18:2	C5:0	FORMYL-L-METHIONYL PEPTIDE
18	LysoPC 14:0		D-SORBITOL
19	LysoPC 16:0		N-ACETYL-D-GLUCOSAMINE
20	LysoPC 17:0		NALPHA-ACETYL-L-LYSINE
21			N-METHYL-D-ASPARTIC ACID
22			PHENYL ACETATE
23			3-HYDROXYKYNURENINE
24			N4-ACETYLCYTIDINE
25			C-GLYCOSYLTRYPTOPHAN
26			ISOPUTREANINE
27			KYNURENIC ACID
28			PUTRESCINE
29			C4 DC
30			C9
31			THEOPHYLLINE
32			PARAXANTHINE
33			CORTICOSTERONE
34			CORTISOL
35			LIPOAMIDE
36			D-GULONIC ACID GAMA-LACTONE
37			L-TRYPTOPHAN
38			INDOLE-3-ACETIC ACID
39			2-3-DIHYDROXYBENZOATE
40			4-AMINOBENZOATE
41			ANTHRANILATE
42			GUANIDINOACETATE

Table S2. The table shows the main metabolic phenotypes and metabolites belonging to each phenotype since phenotype 4 had only one metabolite it has not been included in the analysis

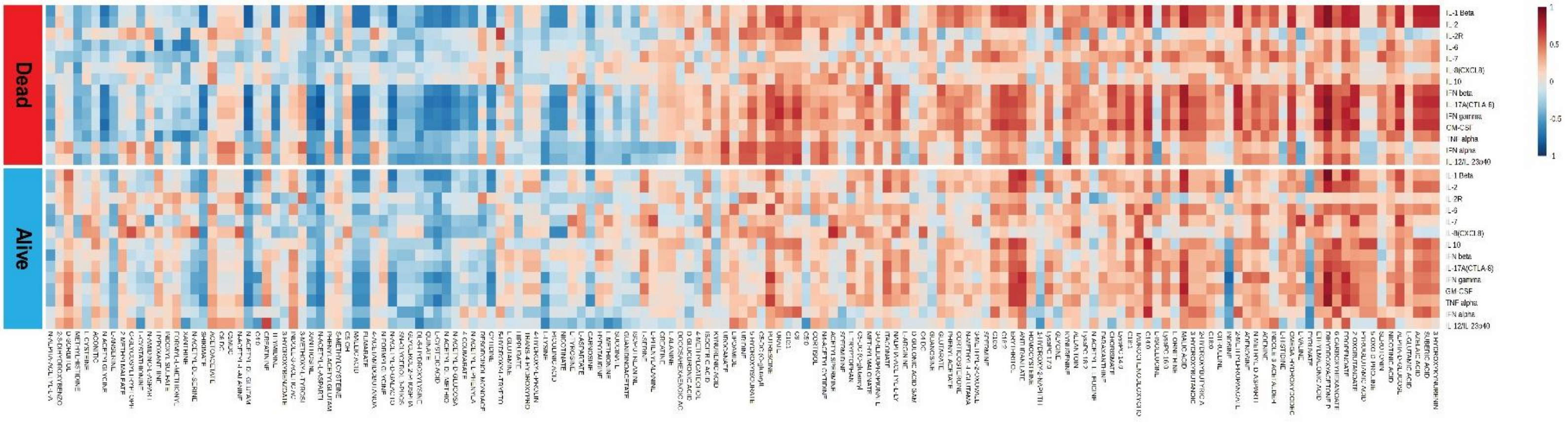

Figure S3. Correlation analysis reveals the correlation coefficients between all metabolites and 14 cytokines of COVID-19 survivors and non-survivors. The figure demonstrates significantly higher correlations of IL-1β, IL-2, IFN-β, IFN-γ, IL-17, and GM-CSF with most metabolites in non-survivors compared to survivors.

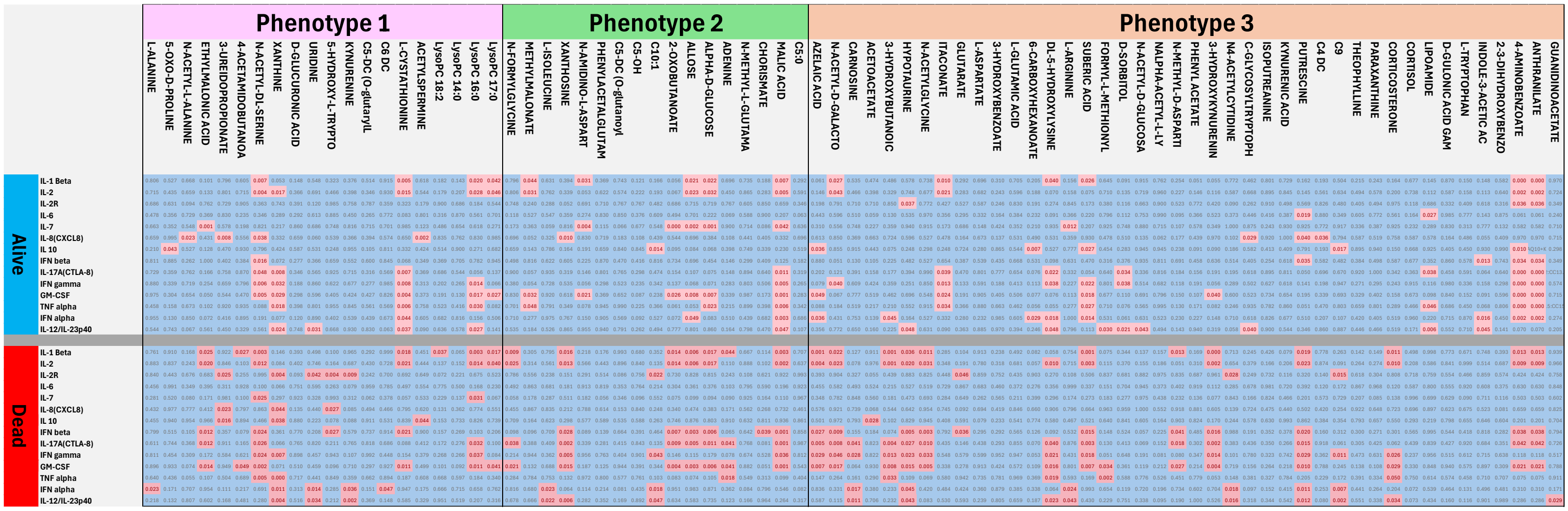

Figure S4. This figure shows the correlation analysis based on the p-value of three metabolic phenotypes with cytokines. Overall, the number of significant correlations between cytokines and metabolites among COVID-19 dead was higher than that among COVID-19 alive.

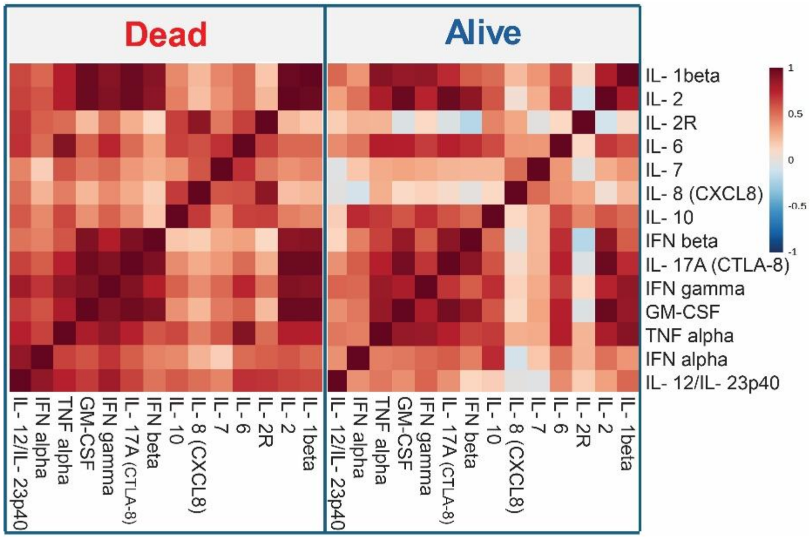

Figure S5. Correlation analysis reveals the correlation coefficients among cytokines in non-survivors and survivors, highlighting distinct correlation patterns, especially for IL-8 and IL-2R among survivors.

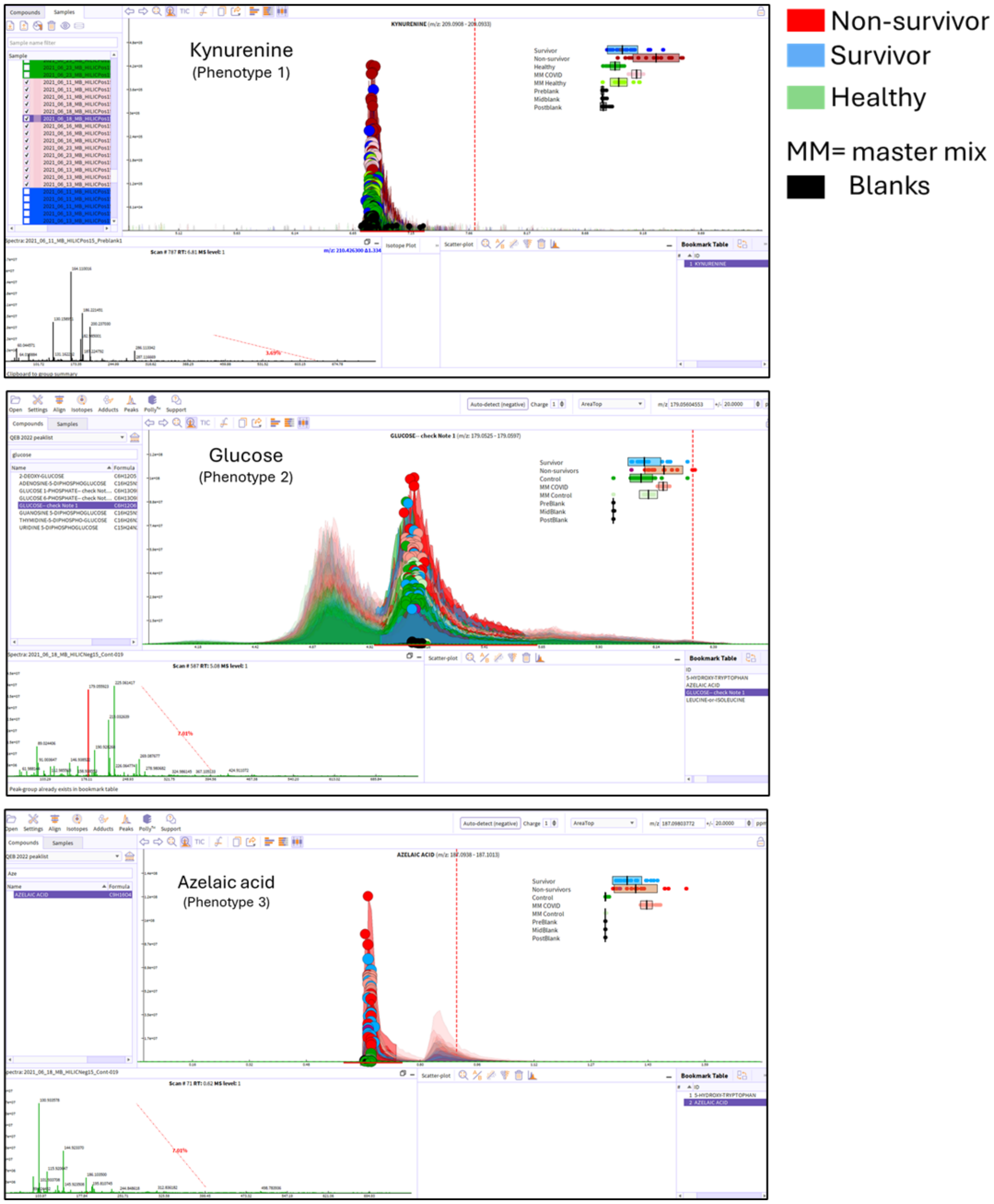

Figure S6. Mass spectrometer chromatograms for one example of each metabolic phenotype.
